# Posterior interhemispheric occipital transtentorial approach for resection of a falcotentorial meningioma

**DOI:** 10.3171/2021.4.FOCVID2125

**Published:** 2021-07-01

**Authors:** Visish M. Srinivasan, Joshua S. Catapano, John P. Sheehy, Mohamed A. Labib, Michael T. Lawton

**Affiliations:** Department of Neurosurgery, Barrow Neurological Institute, St. Joseph’s Hospital and Medical Center, Phoenix, Arizona

**Keywords:** falcotentorial, interhemispheric, meningiomas, occipital, surgery

## Abstract

Falcotentorial meningiomas arise along the junction of the falx cerebri and the tentorium cerebelli. The authors present a woman in her 60s with an incidentally discovered falcotentorial meningioma, approximately 3 cm in diameter, resected with a torcular craniotomy and posterior interhemispheric approach. The galenic complex was dissected away from the tumor. In the final view, the bilateral internal cerebral veins and basal veins of Rosenthal were seen. A Simpson grade I resection was achieved. The patient experienced transient contralateral hemianopsia and was discharged home. At 1-year follow-up, her neurological examination findings were unremarkable, and there was no radiographic evidence of tumor.

The video can be found here: https://stream.cadmore.media/r10.3171/2021.4.FOCVID2125.

## Transcript

This video will demonstrate the posterior interhemispheric occipital transtentorial approach for resection of falcotentorial meningioma.

### 0:30 Presentation

The patient was in her mid-60s. She presented with a tumor that was found incidentally during workup for a transient ischemic attack. She had a history of mild headaches. Her medical history was notable only for breast cancer and a prior smoking history. Neurological examination was completely normal.

### 0:49 Neuroimaging

These are her MRI findings. You can see on axial, coronal, and sagittal gadolinium-enhanced MR images this 3-cm-diameter tumor located squarely at the falcotentorial junction. This CT angiogram shows how the deep venous system, specifically the internal cerebral vein, is occluded by the tumor on its inferior aspect. The straight sinus reconstitutes distal to the tumor and drains to the torcular region. Surgery was recommended, and the patient decided to proceed.

### 1:24 Positioning

She was positioned supine with the head turned to the right. The head was positioned so that gravity retracted the right parietal and occipital regions. The image to the right shows the L-shaped skin incision and biparieto-occipital bone flap that was taken down to the torcular region.

### 1:44 Surgical Strategy

Surgical strategy consisted of the following. Her head was positioned to allow gravity to retract the right parietal lobe. An L-shaped skin incision was made to allow room for this large craniotomy. The craniotomy was made in two pieces. First, a parietal craniotomy to ensure that the dura was nicely preserved, followed by a bilateral extension across the superior sagittal sinus after first dissecting the sinus off of the inner table of bone. Next, a posterior interhemispheric approach was taken down to the quadrigeminal cistern. Transfalcine and transtentorial dissection was performed. Tumor was debulked next. And lastly, the vein of Galen and straight sinus were skeletonized to remove the last portions of tumor.

### 2:34 Operative Video

Here is a view of the operative corridor. The top of the patient’s head is to the right, the torcula is to the left, and the dural flap is rotated over the sagittal sinus in the midline. The posterior interhemispheric fissure begins by detaching these arachnoidal adhesions between the medial hemisphere and the falx. The dissection continues down along the falx, all the way to the region of the quadrigeminal cistern. Here the tumor is encountered for the first time, and you can see its base on the falcotentorial junction. Here the dissection continues distal to the tumor, and you can see branches of the posterior cerebral artery beyond it. Next, the falx and tentorium are cauterized and incised. This enables the arterial base of the tumor to be devascularized. You can see that the falx has been completely transected, reaching the contralateral interhemispheric fissure, and you can see widening of the leaflets of the falx as it joins at the falcotentorial junction. There are inevitably these venous sinuses and channels within these leaflets of dura, and these are carefully coagulated. The incision of the falx is continued posteriorly, skeletonizing the straight sinus, staying on top of the course of the straight sinus. You can see the falx now extending just below the falcotentorial junction. Here the leaflets of the tentorium are being cut, again, to help dearterialize feeders that are coming to the tumor through the tentorium on the contralateral side. You can see how our transfalcine window allows the tumor to fall and for us to reach across very easily.

### 4:24 Debulking

Now the tumor is entered centrally, and it is cored out with an ultrasonic aspirating device. This nicely debulks the tumor and allows the dissection to be developed on the contralateral side. As the tumor is debulked, this nice plane in the arachnoid spaces is developed and the posterior cerebral vessels on the opposite side are freed from their adhesions to the tumor. The falx and tentorium are cut further. The tumor starts to mobilize from the left side downward into the midline corridor. And you can see how there is a very adherent plane on this contralateral side more anteriorly. Therefore, the tumor was aggressively debulked, enabling the capsule to fold inward on itself. This helps to develop this separation plane. Having dearterialized the tumor early with these incisions in the flax and tentorium, the tumor is very dry, and bleeding is easily controlled. Here the dissection continues across the midline to the contralateral side, freeing these adhesions between the contralateral parietal and occipital lobes and the tumor. Now the tumor is being lifted to get across the ipsilateral plane. The tumor is being resected piecemeal. This debulks the tumor and reduces it to just what remains on the deep cerebral veins and the straight sinus. The skeletonization continues until all that remains is the normal venous anatomy. The posterior third ventricle is entered here, between the internal cerebral veins. The deep venous system is inspected. The internal cerebral veins course along the entry into the third ventricle, draining into the vein of Galen to the left. The straight sinus is obscured but is present in the foreground. Here is an overview at the end showing how gravity helps to open the posterior interhemispheric fissure without fixed retraction.

### 6:51 Falcotentorial Meningiomas

Falcotentorial meningiomas are rare. They often compromise the straight sinus and other deep veins, and preoperative venous imaging helps to evaluate that beforehand.^1^ The arterial supply is derived from tentorial arteries: the artery of Davidoff and Schechter, the artery of Wollschlaeger and Wollschlaeger, and the artery of Bernasconi and Cassinari.^2,3^

### 7:16 Outcome

The patient had a transient left homonymous hemianopsia. This improved during her hospital stay and was gone by follow-up examination. Her final pathology was a meningioma, World Health Organization grade 1. At 1-year follow-up, she remained with no evidence of recurrence and no neurological symptoms. Here are her postoperative imaging studies, which demonstrate complete resection of this tumor and preservation of flow in the straight sinus. Here are more postoperative images that show patency of the deep cerebral veins and continuity with the galenic system and straight sinus.

### 7:57 Conclusion

In conclusion, falcotentorial meningiomas are uncommon. They should be differentiated from other tumors in the region, like those involving the posterior third ventricle or pineal region.^4^ The posterior interhemispheric approach reaches the quadrigeminal cistern for early CSF release, and therefore no lumbar drain is needed. Gravity retraction is a useful adjunct that widens the interhemispheric corridor.^5^ Resection of the affected falx and tentorium dearterializes the tumor and enables complete tumor resection. It is important to note that occipital retraction, even without a fixed retractor, can lead to transient hemianopsia postoperatively. Thank you.

## Acknowledgments

We thank the staff of Neuroscience Publications at Barrow Neurological Institute for assistance with manuscript and video preparation.

## Author Contributions

Primary surgeon: Lawton. Assistant surgeon: Catapano, Sheehy. Editing and drafting the video and abstract: Srinivasan, Catapano, Sheehy, Labib. Critically revising the work: Lawton, Srinivasan, Catapano. Reviewed submitted version of the work: Lawton, Srinivasan, Catapano. Approved the final version of the work on behalf of all authors: Lawton. Supervision: Lawton.
